# Mate choice, reproductive success and inbreeding in white rhinoceros: New insights for conservation management

**DOI:** 10.1111/eva.12894

**Published:** 2019-12-16

**Authors:** Petra Kretzschmar, Hailie Auld, Peter Boag, Udo Gansloßer, Candace Scott, Peter John Van Coeverden de Groot, Alexandre Courtiol

**Affiliations:** ^1^ Department of Evolutionary Ecology Leibniz Institute for Zoo and Wildlife Research Berlin Germany; ^2^ Department of Biology Queen's University Kingston Ontario Canada; ^3^ Zoological Institute and Museum of Greifswald University Greifswald Germany; ^4^ Institute of Zoology and Evolutionary Research Friedrich Schiller University Jena Germany; ^5^ Department of Applied Sciences and Computing St. Lawrence College Kingston Ontario Canada; ^6^ Department of Evolutionary Genetics Leibniz Institute for Zoo and Wildlife Research Berlin Germany

**Keywords:** conservation management, inbreeding, mate choice, mating success, paternity assignment, reproductive success, white rhinos

## Abstract

Improving our sparse knowledge of the mating and reproductive behaviour of white rhinoceros (*Ceratotherium simum* Burchell, 1817) is essential for the effective conservation of this iconic species. By combining morphological, physiological and habitat data with paternity assignments of 104 known mother–offspring pairs collected over a period of 13 years, we provide the most comprehensive analysis of the mating system in this species. We show that while the overall mating system was promiscuous, and both males and females produced more offspring when mating with several partners, half of all females with multiple offspring were monogamous. Additionally, we find that mating and reproductive success varied significantly among territorial males in two independent sets of males. In females, however, variation in the mating and the reproductive success was not larger than expected by random demographic fluctuations. Horn size, testosterone metabolite concentration, territory size, habitat openness and the volume of preferred food within the territory did not seem to influence male mating or reproductive success. Moreover, there was no sign of inbreeding avoidance: females tended to mate more frequently with closely related males, and one daughter produced a progeny with her father. The lack of inbreeding avoidance, in combination with the skew in male reproductive success, the partial monogamy in females and the territorial‐based mating system, jeopardizes the already low genetic variation in the species. Considering that the majority of populations are restricted to fenced reserves and private farms, we recommend taking preventive measures that aim to reduce inbreeding in white rhinoceros. A video abstract can be viewed here.

## 1 INTRODUCTION

The world is in a period of mass extinction (Ceballos, Ehrlich, & Dirzo, 2017) with species disappearing at an accelerating pace. Large terrestrial herbivores are particularly affected by this decline (Cardillo et al., 2005; Ripple et al., 2015). Poaching and habitat destruction combined with a slow reproduction rate has resulted in a dramatic decimation of their worldwide populations (Ripple et al., 2015). Africa alone has lost more than half of its large herbivore population within the last 35 years (Craigie et al., 2010). As a consequence, wild herbivores are isolated in islands of protected areas, which are only fractions of their historical range (Ripple et al., 2015). These populations encounter a number of risks if they remain isolated for prolonged periods of time: first, they undergo a loss of genetic variability due to genetic drift, which decreases the fitness of individuals in the short term and leads to a lack of adaptive flexibility in the long term (Frankham, Briscoe, & Ballou, 2002; Giglio, Ivy, Jones, & Latch, 2016; Ralls, Brugger, & Ballou, 1979); second, inbreeding tends to increase due to the limited mating opportunities and thus reinforces the loss of genetic variability; and finally, the lack of gene flow between several small populations of a species leads to divergence in allelic diversity, which decreases future possibilities of cross‐breeding between populations (Brekke, Bennett, Santure, & Ewen, 2010). One of the major challenges in the conservation of isolated populations is to identify the risks that individual populations are facing and to minimize these risks by managing interventions.

The breeding system of a species plays here an important role, as it directly affects the genetic variability of populations (Frankham, 2010; Lande, 1988; Møller & Legendre, 2001; Quader, 2005). It is therefore surprising that the breeding system currently plays only a minor role in the conservation management of isolated populations (Doyle, Hacking, Willoughby, Sundaram, & DeWoody, 2015; Frankham, 2010; Møller & Legendre, 2001; Ralls et al., 2018). In particular, mate choice is a key element in the reproductive behaviour of a species (Anthony & Blumstein, 2000; Kokko & Brooks, 2003). It can cause a bias in the breeding sex ratio and lead to a reduction in the effective population size (Møller & Legendre, 2001; Quader, 2005), while mate choice that avoids closely related individuals contributes to maintaining a genetically diverse population, and can decrease the probability of extinction (Frankham, 1995; Pusey & Wolf, 1996). Mate choice occurs prominently in species with high parental investment (Edward & Chapman, 2011; Trivers, 1972), where the parent investing more in the offspring (usually females) is typically more selective, while the other parent (usually males) rather competes for access to mates, territories, resources or social rank, and evolves traits that affect its competitive abilities (Clutton‐Brock, Harvey, Eisenberg, Kleiman, 1983). Information about the breeding systems and in particular the role that individual traits are playing in mate selection can be used by wildlife managers to define the risk of extinction of individual populations and to identify dominant breeders in their population.

The white rhinoceros is a typical large‐bodied grazer, which was historically distributed in grassland areas all over Southern Africa (Moodley et al., 2018). At the beginning of the 20th century, its worldwide population was decimated by colonial hunters, leaving behind around 100 individuals that survived in a small area at the east coast of South Africa, which was later declared as the Hluhluwe–IMfolozi National Park (Player & Feely, 1960). Following intensive protection, the population recovered and individuals were translocated to various national parks, international zoos and private game farms (Player & Feely, 1960). Today, South Africa conserves 90% of the worldwide white rhinoceros population (Ferreira et al., 2017) and holds a total of around 20,000 individuals (Knight, 2017). Most of these rhinoceros populations are managed, with individuals sold and exchanged between national parks and private game farms on a regular basis. Even the original founder population, in the Hluhluwe–IMfolozi National Park in South Africa, is managed on a yearly basis, and surplus animals are being sold on yearly auctions (de Beer, 2018).

The genetic bottleneck experienced by the white rhinoceros during the colonial period combined with prehistoric declines during the Holocene (Moodley et al., 2018) results in white rhinoceros having a much lower genetic variability than any other rhinoceros species (Guerier, Bishop, Crawford, Schmidt‐Künzel, & Stratford, 2012). Today, the white rhinoceros may be facing another population bottleneck with the ongoing poaching for their horns, which has resulted in the loss of an estimated 8,000 rhinoceros in the past 10 years in South Africa (Department of Environmental Affairs, 2019). The populations holding the largest number of the original founder individuals, the Kruger National Park and the Hluhluwe–IMfolozi National Park have suffered the highest losses. Poaching not only reduces the worldwide rhinoceros population, but also compromises the evolutionary potential of the species by eliminating rare alleles.

Since national parks in South Africa have been less successful in mitigating the effect of poaching than private game reserves, the latter could become the last refuges for the species. They hold a third of the worldwide rhinoceros population (PROA, 2019) and have higher budgets for the protection of rhinoceros than state‐owned conservation areas. Private game farms hold on average around 10 rhinoceros per property (Castley & Hall‐Martin, 2003), with the largest game farms holding more than 1,700 individuals (Stoddard, 2019). The farms are surrounded by fences, preventing dispersal and immigration of new individuals. In the absence of gene flow, these populations of white rhinoceros need careful management in order to best preserve the genetic diversity of the species. However, game farmers often pay little attention to genetic diversity. They remove or introduce new rhinoceros into their population according to the size of the horns or the origin of the new animals, without taking the genetic makeup of an individual into account.

Some wild white rhinoceros already show genetic drift occurring between populations (Moodley et al., 2018), and it is likely that this drift is even more pronounced on private game farms, which usually hold smaller populations compared to conservation areas. This emphasizes that farms and national parks need active management of their remaining populations and that existing guidelines on how to select individual animals for translocation and reintroduction (Emslie, Amin, & Kock, 2009; Emslie & Brooks, 1999) should be expanded to best preserve the genetic variation in the species.

Information about the breeding behaviour of the white rhinoceros is scarce and mainly based on observations in the field (Owen‐Smith, 1975; Rachlow, Berkeley, & Berger, 1998). Such behavioural observations are often sufficient to describe the breeding system of a species, but without genetic confirmation, they can lead to a false characterisation, as it has been shown in a variety of mammals (black rhinoceros: Garnier, Bruford, & Goossens, 2001; seals: Gemmell, Burg, Boyd, & Amos, 2001; white‐tailed deer: Sorin, 2004). Only a combination of genetic paternity assignments together with long‐term behavioural observations allows a comprehensive investigation of mating systems (Clutton‐Brock & Sheldon, 2010).

So far, it is known that white rhinoceros have a territorial‐based mating system, where adult males, which are old and strong enough to defend their own territory, dominate all mating activities. Young sexually mature males between 7 and 9 years of age do not defend a territory and are therefore believed not to participate in breeding (Owen‐Smith, 1975; Rachlow et al., 1998). It is not known whether females further discriminate among territorial males and selectively mate with a territorial owner that is characterized by certain male and habitat characteristics or other traits such as relatedness.

Females range freely between male territories (Owen‐Smith, 1975) and have thus the possibility to discriminate between the different territorial owners. During oestrus, they are closely guarded by males, which try to prevent them from leaving their territory (Owen‐Smith, 1975). Mate guarding can result in aggressive confrontations at the territory border (Owen‐Smith, 1975), which gives the females the possibility to assess the fighting abilities of a male and to choose their mating partners accordingly.

Additionally, females invest substantially into their offspring, while males provide little. Females have gestation periods of 16 months, followed by the birth of calves of ~65 kg, which are nursed for up to 18 months (Dittrich, 1972). In contrast, males join receptive females about 2 weeks before mating and leave them a few days after insemination (Owen‐Smith, 1975). These observations suggest that female white rhinoceros have both the ultimate incentive and proximate opportunities to discriminate between males and that female mate choice, and consequently variation in male reproductive success, could be pronounced in this species.

Several traits are particularly likely to influence mate choice in rhinoceros. First of all, females may select males based on the characteristics of their horns. Indeed, horns and antlers are typical traits that play a direct role in the contest over females (Anderson, 1994). They are efficient weapons and have been correlated with reproductive success in a variety of species, including red deer (Clutton‐Brock, Guinnes, & Albon, 1982; Kruuk et al., 2002), bighorn sheep (Coltman, Festa‐Bianchet, & Jorgenson, 2001) and soay sheep (Preston, Stevenson, Pemberton, Coltman, & Wilson, 2003). Male rhinoceros use their front horn during mate guarding and to defend their territories and the resources therein (Owen‐Smith, 1973, 1975); thick and long horns are here particularly advantageous. For example, males with long horns can more easily injure other rhinoceros without getting in close proximity to their opponent (P. Kretzschmar, pers. observation). Females selecting mates based on their fighting ability could thus confer greater advantages on their offspring (“good genes theory,” Bateson, 1983; “indirect selection,” Kokko, Brooks, Jennions, & Morley, 2003). Second, the androgen level (such as testosterone) is another trait that may be selected by mate choice in rhinoceros. Indeed, androgens control the development of many secondary sexual characters, influence the aggressive motivation and the persistence of aggression, and function, together with cortisol, as a signal of male immune function (Penn & Potts, 1998; Rantala et al., 2012; Wingfield, 2005). Androgens have already been shown to play a role in intersexual conflicts in rhinoceros (Kretzschmar, Gansloßer, & Dehnhard, 2004) and might play a role as an indicator of male quality. Third, females may also select males based on a trait that provide them with direct fitness benefits, such as the quality of the male's territory (“resource defence theory,” Emlen & Oring, 1977; “direct selection,” Kokko et al., 2003). Lastly, females may choose unrelated partners with whom they are genetically more compatible (“genetic incompatibility hypothesis,” Tregenza & Wedell, 2000).

In the following study, we analyse the mating system and mate choice behaviour of the white rhinoceros. We combine 13 years of field observations with genetic paternity assignments of 104 offspring with known mothers. We hypothesize that the mating success and reproductive success of territorial male rhinoceros are influenced by male characteristics such as horn size and testosterone metabolite concentration, by habitat characteristics such as the territory size, habitat openness or grass quality and by relatedness.

Our study was conducted on a private game farm in South Africa, which is housing one of the largest white rhinoceros populations in the country. The population was managed in such a way that all territorial males were exchanged on a 10‐year basis in order to prevent inbreeding. This gave us the opportunity to study the influence of the different traits on the mating and reproductive success of two different groups of territorial males that were introduced sequentially—with removal of the first set of males—into the same population of females. The population was further managed in such a way that all subadult and young adult males were removed in order to prevent lethal fighting between adolescent and territorial males. This management intervention is required when rhinoceros have no possibility to disperse. Our study therefore describes the mating system and mate choice behaviour of a managed population of white rhinoceros, which is representative of most isolated rhinoceros populations in Southern Africa.

## 2 METHODS

### 2.1 Study site and population

The study was conducted on a 300‐km^2^ privately owned game farm in the Limpopo Province, South Africa. The fenced reserve is categorized as typical savannah at a certain stage of degradation (EEC, 1998). The climate is defined by wet summers and dry winter with an average annual rainfall of 464 mm (Kretzschmar et al., 2004).

The white rhinoceros population was founded in 1990 with 29 individuals originating from three different locations in South Africa. At the beginning of the study, in March 1997, the population housed a total of 61 individuals (23 males and 38 females), including six territorial males and eight subadult males. The population density at that time was 0.23 km^2^. This is comparable to other rhinoceros populations such as in the Kruger National Park (0.5–1.4 km^2^; Pienaar, Bothma, & Theron, 1993) or the Ndumu Game Reserve (0.6–1.8 km^2^; Conway & Goodman, 1989). In the study, we used the term “territorial males” to describe all males over 10 years of age that defend a territory. We further distinguished between “young adults” that were sexually mature but did not defend a territory (7–9 years old) and “subadult males” that were not yet sexually mature (3–6 years old). Subdominant males, which have been described in areas of high population density, such as in the Hluhluwe–IMfolozi National Park (3–6 rhinoceros per km^2^; Owen‐Smith, 1975), have not been observed in our study.

All territorial males present on the reserve from the beginning of the study until July 2001 are referred to as cohort 1 (C1, male “A,” “G,” “K,” “R,” “S”), plus one adult male “123” that died in June 1997 during a territorial fight. He was included in the parentage analysis and in the study on relatedness, but, due to the lack of data, in no further analysis. In July 2001, all territorial males (C1, *n* = 5) were removed from the farm and replaced by six new adult males (male “5,” “30,” “62,” “63,” “65” and “66”; referred to as cohort 2 or C2) and one young adult male (male “60” of around 7 years of age). This exchange was conducted in order to prevent inbreeding and enabled us to study the characteristics and the reproductive success of two groups of territorial males. The new males originated from four different locations in South Africa. The young adult male “60” died in 2003 during a territorial fight and never occupied a territory. We used his genetic information for parentage analysis only.

In fenced reserves, rhinoceros are often kept in unnatural high density, which causes aggression. To prevent lethal fighting between adolescent and territorial males, all subadult and young adult males were removed from our study population, with the exception of male “60.” The removal started in July 1998 and was repeated once every year. The remaining population thus consisted only of adult territorial males and females with their young at foot.

### 2.2 Data collection

Data collection started in March 1997. During the first 2 years (until May 1999), individual males of the first cohort (C1) were tracked on foot until they have been sighted with the help of an experienced game tracker. Each male was located approximately twice per week, and its final position was established using a Global Positioning System (GPS, mean ± standard deviation = 128 ± 27 locations/male). The males from the second cohort (C2) were observed for a longer time period, from July 2001 until December 2008, but less frequently. Their location was established approximately once per month, by rangers from sightings during the daily patrols (mean = 83 ± 26 locations/male). These sightings were supplemented with information from rangers providing GPS locations of individually known dung heaps, foot path and fighting events (*n* = 18 ± 6 locations/male). Due to the difference in sampling protocol, comparing the size of the territories between the two cohorts of males would not be meaningful.

Tissue collection started in 1998 when all animals were captured, anaesthetized, measured and ear‐notched. Find further information about data collection and notching in the video abstract here. The ear notching was repeated on a yearly basis with the capture of all animals between 1 and 2 years of age. We thus managed to collect a complete set of skin samples of all offspring and their parents, including juveniles that were up to 3 years of age during the first notching event and not weaned from their mothers yet, which usually takes place around 3 years of age (Owen‐Smith, 1975). Our study thus includes mother–offspring relationships collected over a time period of 13 years.

### 2.3 Male characteristics

A territorial male was described using the size of his horns and the concentration of the testosterone in his faeces as indicators of male quality.

#### 2.3.1 Horn size

We measured the length and the circumference of the anterior and posterior horn along the anterior curve and along the base from tranquillized males (for C1 in 1998 and for C2 in the year 2001).

#### 2.3.2 Testosterone metabolite concentration

Faecal samples from the males (C1: mean = 59 ± 6 samples/male; C2: mean = 5 ± 0.4 samples/male) were collected during tracking, when the sample was fresh and its origin was known, and when we sighted an excreting animal. Additional samples (*n* = 2 per male) were obtained from the rectum of the anaesthetized rhinoceros. The number of samples per individual and months was carefully balanced (C1: mean = 5 ± 2 samples per male and month collected between May 1997 and April 1999; C2: mean = 2 ± 0.2 samples per male and month collected between August and September 2001) in order to capture the seasonal variation in hormone concentrations (Kretzschmar et al., 2004). Due to the difference in the time of sampling between both cohorts of males, comparing the testosterone metabolite concentrations between the two cohorts of males would not be meaningful.

Faecal samples from different parts of the dung heap were mixed in a plastic bag and stored (0.5 g) with 5 ml methanol (90%) at −12°C. The samples were processed and analysed using an antibody against 17α‐OH‐testosterone‐HS‐BSA and an enzyme immunoassay (EIA) as described in Kretzschmar et al. (2004). Faecal hormone concentrations were expressed as ng/g dry weight.

### 2.4 Habitat characteristics

The territory size was used as an indicator of the abundance of resources therein, while habitat openness was used as a negative measure of shelter and the volume of selected food as a positive measure of food quality.

#### 2.4.1 Territory size

The territory size was established using the GPS positions recorded for C1 and C2 males. A maximum of two GPS locations per day were used, providing that they were 6 hr apart to limit autocorrelation. A polygon was drawn around the outer locations using Quantum Geographic Information System (QGIS) (Open Source Geospatial Foundation Project; version 1.7.4.). This method enabled us to describe biological meaningful territory borders as established during field surveys and to exclude areas outside of the reserve. For male “S” of C1, who lost his territory during the study period, locations before he was displaced were used. All locations collected during the first 3 months after reintroduction of the C2 males were removed from the analysis since they are likely reflecting the exploration of the new habitats and not the final territories.

#### 2.4.2 Habitat openness

Habitat openness was assessed at transect points distributed evenly over the entire study area (*n* = 143, S1). The openness of a habitat was visually assessed by comparing pictures of predefined categories with the surrounding of the transect point. Four types were identified: “grassland” with a visibility greater than 50 m and the absence of canopy cover and understory, and three types of “woodlands” including trees and understory in varying degrees ranging from a visibility of approximately 50 m (“open woodland”), to 25 m (“close woodland”) and <25 m (“thickets”). We established the frequency of each category of openness in relation to the total number of transect points within each territory.

#### 2.4.3 Volume of selected food

As a first step, we conducted a study on food selection. We followed the footprints of foraging females (*n* = 6) with the help of a local game tracker and identified grass species that have been eaten by them. We then calculated the frequency of a grass species that has been eaten in relation to its availability (S2). The volume of selected grass species within male territories was established by measuring the cover and average height of grass species along transect points (Greig‐Smith, 1957, S2). Height and cover were then multiplied to establish the volume of each grass species. A mean volume per territory was calculated from measurements located at transect points within the territory boundaries.

### 2.5 DNA extraction and microsatellite genotyping

A total of 154 tissue samples were collected, comprising 104 offsprings sired over a period of 13 years (1995 till 2008), 37 mothers  and 13 candidate males (C1 and C2 males, including male “123” and male “60,” which potentially fathered offspring during the short time they were present).

Samples were collected by registered veterinarians, exported with required permits under CITES and imported according to the regulations of the Canadian Food Inspection Agency (CFIA). The samples were stored in 90% ethanol, and DNA was extracted using a standard phenol–chloroform extraction (Maniatis, Fritsch, & Sambrook, 1982). The microsatellites were amplified by polymerase chain reaction (PCR) and sequenced using a LI‐COR 4200 detection system (Scott, 2008).

We initially genotyped all tissue samples at 11 microsatellite loci developed for different rhinoceros species: WR32A, WR32F and WR35A (Florescu et al., 2003); SR54, SR63, SR262 and SR281 (Scott et al., 2004); BR06 (Cunningham, Harley, & O'Ryan, 1999); DB01 and DB44 (Brown & Houlden, 1999); and IR12 (Scott, 2008; see Table S1 for full specification). Each locus was tested for the presence of null alleles and stutter errors using Micro‐checker (Van Oosterhout, Hutchinson, Wills, & Shipley, 2004) and deviations from the Hardy–Weinberg equilibrium using Genetic Analysis in Excel (GenAlEx, Peakall & Smouse, 2006; Table S1). The locus WR32F deviated significantly from the Hardy–Weinberg expectations (HWE, *p *< .001) and was discarded from subsequent analyses. Observed (*H*_o_) and expected (*H*_e_) heterozygosities and polymorphic information content (PIC) were calculated using CERVUS 3.0.7 (Kalinowski, Taper, & Marshall, 2007; Marshall, Slate, Kruuk, & Pemberton, 1998; Table S1). We determined mean locus‐specific genotyping error by repeating five PCR for a subset of 50 randomly chosen individuals and calculated the number of times replicate genotypes differed from the most common genotype for that individual (Tables S1 and S3).

### 2.6 Parentage analysis

We used sibship reconstruction in COLONY 2.0 (Jones & Wang, 2010) to assign paternities and to study the reliability of such assignments using simulations. COLONY uses maximum‐likelihood methods to partition offspring genotypes into maternal and paternal full‐ and half‐sibships (Jones & Wang, 2010; Wang, 2004).

Following Wang (2013), simulations were used to choose the most appropriate method to analyse our data and to assess the accuracy of the parentage assignment. Specifically, we used simulations to compare three different scenarios to perform the paternity assignment: in scenario 1, we included all candidate parents in a single data set and created a mating matrix with 13 potential fathers and 33 potential mothers defining the rows and columns of the matrix, respectively. The number of potential mothers and their offspring was based on a preliminary assignment of parentage from our data set. We filled the cells of the matrix using 68 offspring, which were distributed in the matrix similar to the empirical data set. In scenario 2, we used the same mating matrix and added a matrix defining the known mother–offspring relationships. In this, the simulation only aims at assigning fathers. In scenario 3, we decided to split the data into the two male cohorts and ran a separate analysis for each, considering again the known mother–offspring relationships. The two mating matrices in this scenario thus included the adult females that were present until males were introduced or removed from the study site (*n* = 28 for C1 males, *n* = 23 for C2 males) and the offspring that had been assigned to a father in a preliminary parentage analysis and that had been sired up to 16 months (average gestation time in rhinoceros; Dittrich, 1972) after the removal of C1 males (*n* = 36 for C1 males, *n* = 32 for C2 males).

We ran each of the described simulations using the following settings: long run, high likelihood precision, male and female polygamy, inbreeding without sibship scaling and weak sibship size prior. We defined the average paternal and maternal sibship size based on a preliminary assessment of parentage, for which we obtained a value of 5.6 for males and 1.6 for females. The inbreeding coefficient of the parent was calculated using the maximum‐likelihood estimator of relatedness (see Section 2.7). The probability of both parents being present in the candidate pool was set at 100%, as all mothers and potential fathers were individually known and included in the analysis. We used a full‐likelihood method with updating of the allele frequency and included the allele frequency that we calculated a priori in CERVUS from all mothers, fathers and offspring of the population (*n* = 117). We selected a conservative estimate of 0.01 for allelic dropout, which was based on preliminary parentage assignments with known mother–offspring pairs. 

The power of the parentage assignments (mean and *SD*) was assessed using ten replicate simulations and includes all assignments that reached a confidence level equal or higher than 80%.

The outcome of this simulation analysis revealed that the probability to assign the true father as the most likely father was highest (86.5% ± 11.6), when the number of candidate fathers and mothers is constrained (following scenario 3). Using only the known mother–offspring relationship in the paternity assignment (scenario 2) reduced the chance to assign the true father to 75.2% ± 7, while using no information from the pedigree (scenario 1) resulted in the lowest power to assign the true father (57.9% ± 12).

Based on these results, we ran the actual parentage assignment on all genotyped offspring using the scheme defined as scenario 3. Specifically, we included all C1 males plus “123” (*n* = 7) with all their potential mating partners (*n* = 31) and included all offspring (*n* = 53) born between June 1995 and November 2002. We restricted paternity for male “123,” who died in June 1997, and could not be the father of offspring born after October 1998. In the second run, we included all C2 males plus male “60” (*n* = 7), their potential mating partners (*n* = 32) and their offspring (*n* = 51). We excluded offspring born after November 2004 for male “60,” since he died in July 2003. We ran the paternity assignment using the same settings as described in the simulation and defined the maternity exclusion threshold to 1.

### 2.7 Relatedness

We calculated the degree of relatedness between each C1 (or C2) male and all reproductively active females to test whether relatedness was negatively correlated with mating and reproductive success. Additionally, we calculated the degree of relatedness between all C1 and C2 males and their mating partners (established from paternity data), to establish whether males mated with less related females compared to the available pool of mating partners.

Different marker‐based estimators have been proposed to assess relatedness in wild populations. Following Taylor (2015), we identified the most appropriate metric for our data set by comparing the performance of all seven marker‐based estimators of relatedness available in COANCESTRY (Wang, 2010a). Specifically, we simulated 100 dyads for each of the following six relatedness categories (*n* = 600) using the allele frequency from all parents and offspring in our population (*n* = 117): parent–offspring (PO), full‐siblings (FS), half‐siblings (HS), first cousins (FC), second cousins (SC) and unrelated individuals (U).

The simulation revealed that the maximum‐likelihood estimator of relatedness DyadML (Milligan, 2003) performed best and correctly predicted variation in the true degrees of relatedness simulated, with the exception of second cousin for which the median relatedness was overestimated (Figure S1). Nonetheless, there was a large overlap in the distribution of estimated relatedness between the six relatedness categories simulated.

### 2.8 Mating and reproductive success

We used the parentage information from our genetic analysis to establish the mating and reproductive success of all males (C1, C2) and females (*n* = 37), including the two additional males mentioned above (“123” and “60”). We defined mating success (mat) as the number of breeding partners with which offspring were sired, and reproductive (rep) success as the total number of assigned juveniles (Jones, 2009). We used this information to compute a standardized Bateman gradient (*β*_SS_). This gradient provides the direct fitness benefit to multiple mating by measuring the slope of the linear regressions of relative reproductive success on relative mating success (Jones, 2009). Additionally, we tested whether the mating and the reproductive success varied among the males (C1 + C2) by calculating the Nonac's binominal skew index (Nonac's *B* index; Nonacs, 2000). This index is used to compare the observed variance with the expected binominal variance stemming from demographic stochasticity alone. It accounts for variation in group size, productivity (i.e., mating or reproductive success) and differential residential times between individuals. It has been calculated as:

$$B=\sum_{i=1}^{N}{\left(p_{i}-\frac{n_{i}}{N_{t}}\right)}^{2}-\left(1-\frac{1}{\overset{´}{N}}\right)/K,$$

*N* is the total number of males that were present on the study site, *p_i_* the proportion of the total number of benefits (i.e., number of successful mating or number of juveniles sired by each male), and *n_i_* the time male *i* spent in the group. The cumulative time spent in the group across all individuals is $N_{t}\;=\;\sum_{i=1}^{N}n_{i}.$ This value is also used to compute the weighted mean group size: *Ń* = *N_t_*/*n*_max_, where n_max_ is the maximum time any individual could be present (i.e. 8 years for C1 males, 6 years for C2 males, and 12 years for females).

### 2.9 Statistical analysis

All statistical analyses were performed using the functions readily available in the statistical software R v3.6.1 (R Development Core Team, 2019). We only used additional R packages for plotting purposes (ggplot2 v3.2.1, cowplot v1.0.0 and ggforce v0.3.1).

We tested for significance in variance of mating and reproductive success by comparing the observed variance to the distribution of the Nonac's *B* index simulated under the null hypothesis of stochastic fluctuations alone. For this analysis, the two cohorts of males were pooled. The horn measurements of each cohort of males were converted into a single composite variable using a principal component analysis (PCA, function *prcomp* in R). The resulting variable—hereafter referred to as “horn characteristics”—was then used to describe the morphology of the horns of each male.

These variables and the measurements for the testosterone metabolite concentrations, habitat characteristics and relatedness were correlated with the male mating and reproductive success using the Spearman rank correlation test (function *cor.test* with argument *exact* = *FALSE*). This nonparametric test does not require any assumption about the distribution of the data, which we could not assess due to the small number of adult males. For the same reason, we did not attempt to analyse the simultaneous effect of all potential predictors in a generalized linear model. Yet, a conservative Bonferroni correction was applied to all correlation tests, which allows for counteracting the increase in the rate of false positives due to multiple testing. Specifically, we multiplied *p*‐values obtained by the number of tests (*n* = 9) performed within a given male cohort and within a given fitness component (mating or reproductive success). These corrected *p*‐values are referred to as expect values (*E*‐value). We relied on the Fisher exact test (function *fisher.test*) to compare the parentage assignment rates between the two cohort of males and used a Wilcoxon signed rank test (function *wilcox.test* with argument *paired = TRUE*) to test whether males showed a different level of relatedness with females with which they actually mated as compared to all available females.

## 3 RESULTS

### 3.1 Parentage analysis

A total of 151 juveniles were born between March 1995 and December 2008 of which 69% (*n* = 104 juveniles) were sampled and genetically analysed using 10 microsatellite loci (Table S1). Fatherhood was assigned to 70% (*n* = 73) of all father–offspring combinations at the confidence level of 80% or higher.

Paternity was assigned to 64% (*n* = 34) of C1 (*n* = 53) and to 76% (*n* = 39) of C2 offspring (*n* = 51). The assignment rate did not significantly differ between the two cohorts (Fisher's exact test: odds ratio = 0.55, *p* = .20).

### 3.2 Mating and reproductive success

**Figure 1:**
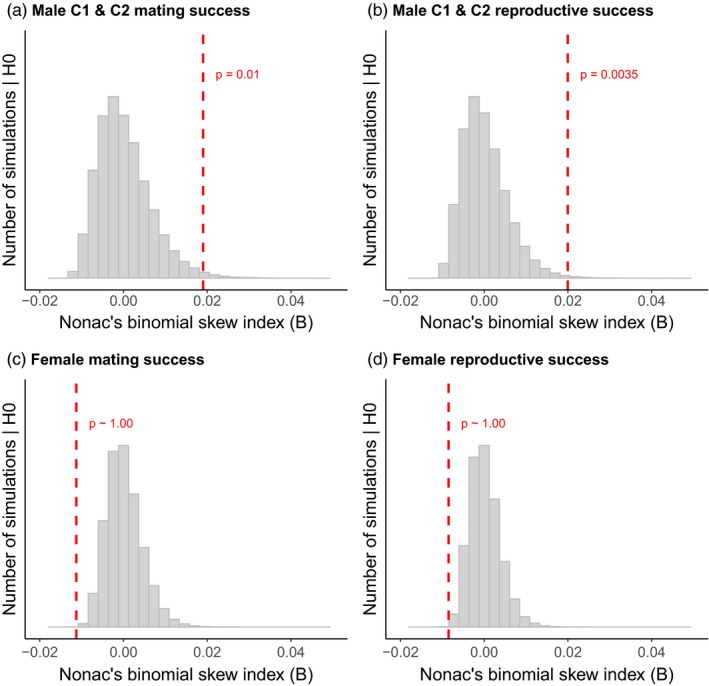
Comparison of the observed variance in mating (a) and reproductive success (b) of males of cohort 1 and cohort 2, as well as mating (c) and reproductive success (d) of all reproductive active females to the corresponding variance expected under the null hypothesis (Nonac's *B* index). The vertical dashed lines indicate the observed amount of variance. The grey bars represent the distribution of the amount of variance simulated under the null hypothesis (*N*_simulations_ = 100,000). The *p*‐values indicate the probability to obtain a value as large as or larger than the one observed under the null hypothesis

**Figure 2:**
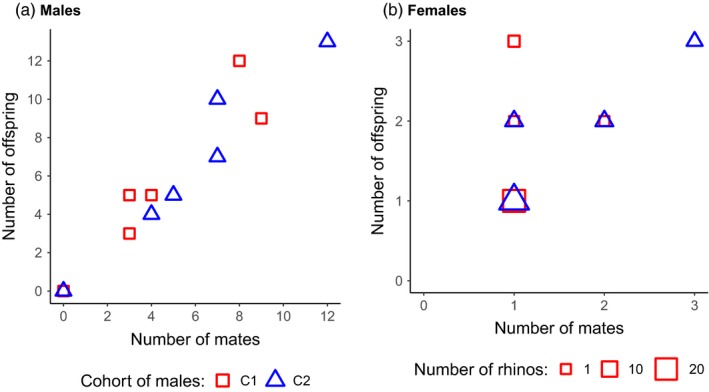
Relationship between the mating success and reproductive success of male (a) and female (b) white rhinoceros. The shape of the symbols indicates whether the fathers of the offspring belong to cohort C1 (squares) or cohort C2 males (triangles). The size of the symbol provides the relative sample size

The mating success (number of breeding partners) and the reproductive success (number of assigned juveniles) significantly varied among candidate males even after accounting for the effect of demographic stochasticity (Nonac's *B* index C1 and C2: *B*_mat_ = 0.019, *p* = .010, *n* = 12, Figure 1a; *B*_rep_ = 0.020, *p *< .0035, *n* = 12, Figure 1b). The reproductive success was positively correlated with an increase in mating success in all males (C1: Spearman's *ρ* = .90, *p* = .015, *n* = 6, Bateman gradient *β*_SS_ = 0.94; C2: *ρ* = .99, *p *< .001, *n* = 6, *β*_SS_ = 1.00; Figure 2a).

Female rhinoceros showed no evidence of skew in mating and reproductive success (*B*_mat_ = −0.011, *p* ~ 1.00, *n* = 33, *B*_rep_ = −0.0085, *p* ~ 1.00, *n* = 33; Figure 1c,d). Their reproductive success was positively correlated with an increase in mating success with C2 males (*ρ* = .75, *p *< .001, *β*_SS_ = 0.79, *n* = 27; Figure 2b), but not with C1 males (*ρ* = .35, *p* = .08, *β*_SS_ = 0.57, *n* = 26). Out of all females (*n* = 16) that had multiple offspring during the presence of a single cohort of males, half of them bred with a single male and produced up to three consecutive offspring with the same partner (Table S2).

Male “K” was the most successful male in cohort 1 (Table S2). He mated with 8 different females and sired 12 offspring (he may have had additional mating partners and offspring which we could not retrieve as not all offspring were successfully assigned to their father). All other males sired between nine and zero calves and mated with nine or less females. In cohort 2, male “65” was the most successful male. He mated with at least 12 females and sired at least 13 calves. The young adult male “60” did not mate with any female and was not assigned to any calf.

### 3.3 Male characteristics

#### 3.3.1 Horn characteristics

**Figure 3:**
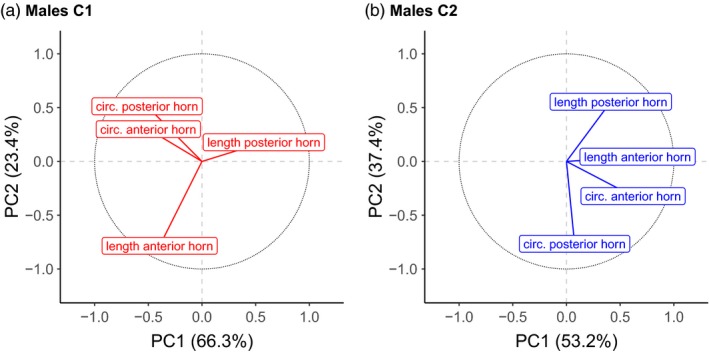
Principal component analysis (PCA) of horn measurements. The figure shows the projection of the four horn measurements on the two main principal components PC1 and PC2 for the two cohorts of males C1 (a) and C2 (b). The principal components explain 66.3% (PC1) and 53.2% (PC2) of the variability of the horn measurements

**Figure 4:**
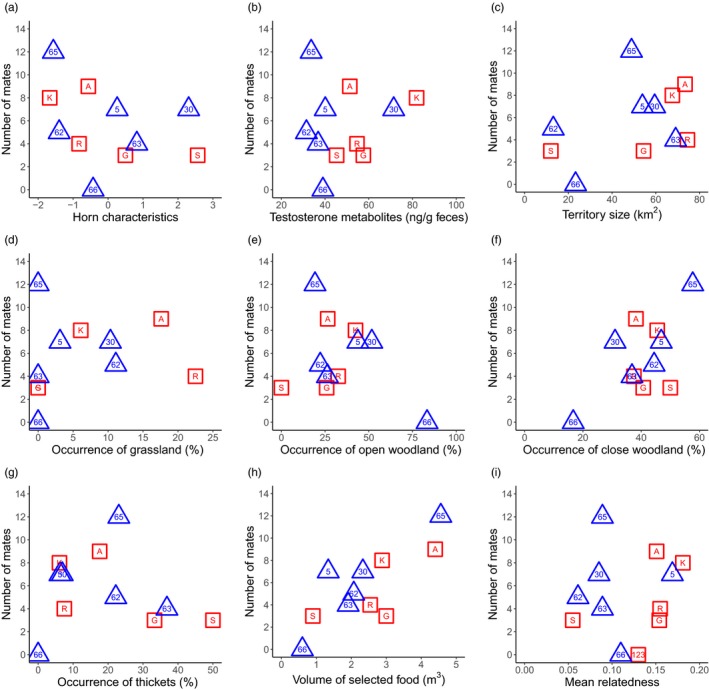
Relationship between the mating success and two male characteristics (a, b), six habitat characteristics (c–h) and relatedness (i) for the two male cohorts. The shape and colour of the symbols refers to the cohort (red square = cohort 1, blue triangle = cohort 2). The labels within symbols refer to the male identifier

**Figure 5:**
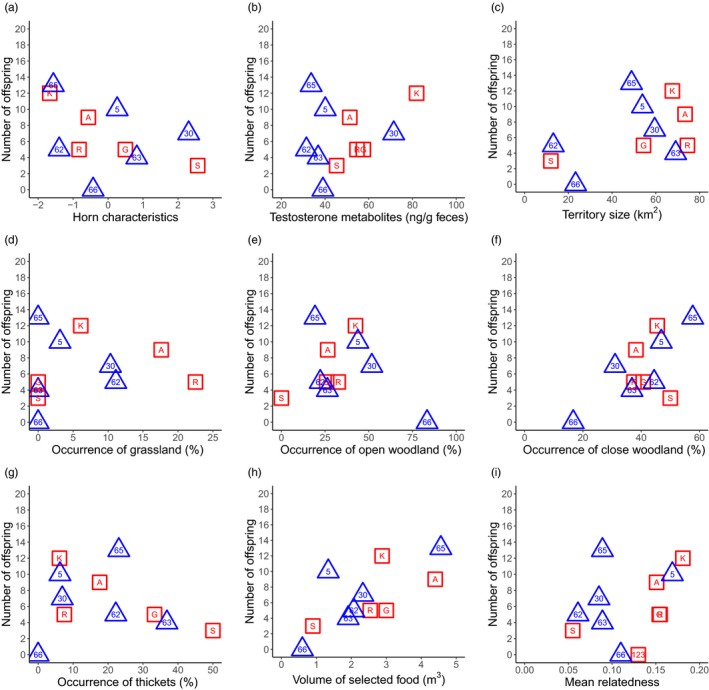
Relationship between the reproductive success and two male characteristics (a, b), six habitat characteristics (c–h) and relatedness (i) for the two male cohorts. The shape and colour of the symbols refers to the cohort (red square = cohort 1, blue triangle = cohort 2). The labels within symbols refer to the male identifier

The first principal component calculated by PCA from the horn size of the males summarized 66.3% (C1), respectively 53.2% (C2) of the total variation of the four horn measurements (Figure 3a,b). Figure 3 shows that the higher the values for the component, the smaller the first horn for C1 males (Figure 3a) and the larger both horns for C2 males (Figure 3b). The mating and reproductive success did not correlate significantly with the horn component in C1 or C2 males (C1_mat_ & C2_mat_: Figure 4a, C1_rep_ & C2_rep_: Figure 5a; see Table 1 for summary of results). The negative correlation between horn size and reproductive success appears relatively strong in C1 males despite the lack of significance before applying the Bonferroni correction (*ρ* = −.82, *p* = .089; Figure 5a), suggesting that males with larger first horn could have reproduced more. Yet, the corresponding negative correlation in males C2 (*ρ* = −.26, *p* = .62) points towards the opposite conclusion due to the changing relationship between the first principal component and the horn dimensions.

**Table 1:** Summary statistics for the Spearman rank correlation test comparing the mating success and reproductive success of cohort 1 and cohort 2 males with the male and habitat characteristics and with relatedness. The *p*‐values before (*p*) and the expect value (*E*) after the Bonferroni correction are provided in the table

	Cohort 1 males							Cohort 2 males						
	*n*	Mating success			Reproductive success			*n*	Mating success			Reproductive success		
		*ρ*	*p*	*E*	*ρ*	*p*	*E*		*ρ*	*p*	*E*	*ρ*	*p*	*E*
Male characteristics														
Horn characteristics	5	−.67	.22	2.0	−.82	.089	0.80	6	−.20	.70	6.3	−.26	.62	5.6
Testosterone metabolite concentration	5	.15	.80	7.2	.67	.22	2.0	6	.058	.91	8.2	.029	.96	8.6
Habitat characteristics														
Territory size	5	.67	.22	2.0	.46	.43	3.9	6	.12	.83	7.4	.086	.87	7.8
Habitat openness														
Grassland	5	.68	.20	1.8	.39	.51	4.6	6	.18	.73	6.5	.15	.77	7.0
Open woodland	5	.67	.22	2.0	.82	.089	0.80	6	−.52	.29	2.6	−.54	.27	2.4
Close woodland	5	−.41	.49	4.4	−.21	.74	6.7	6	.75	.084	0.75	.83	.042	0.37
Thickets	5	−.67	.22	2.0	−.82	.089	0.80	6	.23	.66	5.9	.20	.70	6.3
Volume of selected food	5	.56	.32	2.9	.62	.27	2.4	6	.75	.084	0.75	.66	.16	1.4
Relatedness	6	.52	.29	2.6	.75	.084	0.75	6	−.088	.87	7.8	.029	.96	8.6

#### 3.3.2 Testosterone metabolite concentration

The mean testosterone metabolite concentration for C1 males was 58.1 ± 14.0 ng/g faeces (*n* = 5) and for C2 males 42.1 ± 14.7 ng/g faeces (*n* = 6, Figure S2). The variance in concentration between both cohorts is expected because samples were processed at different times. Despite the variance among cohorts, the concentration of testosterone metabolites was higher in territorial adult males than in nonterritorial adult males (31.3 ng/g faeces; Rachlow et al., 1998), subadults or juveniles (28.5 ng/g faeces; Kretzschmar et al., 2004).

The mating success and reproductive success of C1 and C2 males were not significantly correlated with the mean testosterone metabolite concentrations (C1_mat_ & C2_mat_: Figure 4b, C1_rep_ & C2_rep_: Figure 5b; Table 1).

### 3.4 Habitat characteristics

#### 3.4.1 Territory size

The territories of C1 males ranged from 12.0 km^2^ (male “S”) to 74.5 km^2^ (male “R”). In C2 males, the size of the territories ranged from 13.1 km^2^ (male “62”) to 69.1 km^2^ (male “63”). Mating and reproductive success did not correlate with the territory size of both cohorts (C1_mat_ & C2_mat_: Figure 4c, C1_rep_ & C2_rep_: Figure 5c; Table 1).

#### 3.4.2 Habitat openness

The male territories were composed of all four habitat types, with “close woodland” predominating in the territories of C1 and C2 males (Table S3). The mating success and reproductive success were not significantly correlated with the occurrence of any habitat type (C1_mat_ & C2_mat_: Figure 4d–g; C1_rep_ & C2_rep_: Figure 5d–g; Table 1). The reproductive success of C2 males did correlate significantly with the presence of “close woodland” before applying the Bonferroni correction (*ρ* = .83, *p* = .042; Figure 5f), but the direction of the correlation was not retrieved in C1 males (*ρ* = −.21, *p* = .74).

#### 3.4.3 Volume of selected food

Females foraged in 85% of all cases on *Panicum maximum* (Jacq.) when it was available along their feeding path. The volume of *Panicum maximum* differed between the territories (Table S3), but the differences in volume did not influence the mating and reproductive success of both cohorts (C1_mat_ & C2_mat_: Figure 4h, C1_rep_ & C2_rep_: Figure 5h; Table 1).

### 3.5 Relatedness

Mating and reproductive success did not show a significant negative correlation with the mean relatedness of males to all females in both cohorts, as we would expect under inbreeding avoidance (C1_mat_ & C2_mat_: Figure 4i, C1_rep_ & C2_rep_: Figure 5i; Table 1). In fact, the correlations were either weak or even positive such as in the case of mating or reproductive success and the mean relatedness of C1 males to females (Table 1). Furthermore, the mean relatedness between all males with assigned offspring (*n* = 10) and their mating partners was slightly higher (mean = 0.14 ± 0.06) compared to the mean relatedness of these males and the available females (mean = 0.12 ± 0.05). This indicates that females tend to mate more frequently with closely related males; however, the trend is not significant (Wilcoxon signed rank test, *W* = 44, *p* = .10; Figure S3). In addition, our paternity analysis assigned a daughter to her father (male “A”; Table S2).

## 4 DISCUSSION

We provide the first genetically based assessment of the mating system of the white rhinoceros and identify three key factors that threaten the already low genetic diversity in this species: (a) a promiscuous mating system in which half of all females with multiple offspring are genetically monogamous, (b) a large variance in mating and reproductive success among territorial males and (c) a lack of inbreeding avoidance during mate choice.

Monogamy is infrequent in mammals and usually occurs in socially monogamous species such as in primates or rodents (Lambert, Sabol, & Solomon, 2018; Ophir, Phelps, Sorin, & Wolff, 2008). Genetic monogamy, which describes exclusive reproduction between mating partners without any social relationship (Reichard & Boesch, 2003), has to our knowledge only been described for nonmammal species (Wickler & Seibt, 1983), except for black rhinoceros (Garnier et al., 2001). The lack of evidence in other species might be due to the difficulty to collect individually based data of all members of a population over several breeding seasons. The long study period of 13 years enabled us to assess paternity of up to five offspring produced by an individual female, which resulted in a maximum of three offspring per female and male cohort. Our study thus nearly covers half of the maximal reproductive lifespan of a female white rhinoceros (approximately 34 years; Groves, 1972; Owen‐Smith, 1975) and thus represents the longest study on female mating behaviour in the species. Video abstract can be viewed here.

Our study revealed that males were more promiscuous than females: all males that produced multiple offspring mated with several females. Moreover, we found a strong interindividual variation in both male mating success and reproductive success, which was significantly higher than what is expected from demographic stochasticity alone (Nonacs, 2000). This was not the case for females. The steep Bateman gradient we observed in males and females indicates that both sexes obtained a direct fitness benefit in terms of number of offspring from multiple mating (Jones, 2009). The possibly steeper gradient in males than in females is consistent with other studies on mammals (Janicke, Häderer, Lajeunesse, & Anthes, 2016). Overall, our results thus confirm our assumption that there is strong male–male competition and/or female choice in the species, while female–female competition and/or mate choice exerted by males is weak.

To understand what drives mating behaviour in the white rhinoceros, we tested the influence of several factors that we hypothesized to affect mating and reproductive success: horn characteristics, testosterone metabolites, habitat structure, volume of selected food in the territories and relatedness. Our results show no significant correlation between mating success and reproductive success to any of the analysed traits. We did observe strong correlations between horn characteristics and reproductive success in the first cohort of males (C1), suggesting that males with a long anterior horn could have reproduced more, but the corresponding correlation in a second cohort of males (C2) points towards the opposite conclusion. The idea that females select mates based on their fighting ability (“good gene theory”; Bateson, 1983) or on their ability to secure resources with their horn (“resource defence theory”; Emlen & Oring, 1977) could therefore not be confirmed in this study. We found a significant correlation between reproductive success and the presence of “close woodland” but only before applying the Bonferroni correction, and the direction of the correlation was not retrieved in the other cohort.

Contrary to what would be expected under the “genetic incompatibility hypothesis” (Tregenza & Wedell, 2000), we did not find any strong negative correlation between the mating or reproductive success and the relatedness measured between the males and all candidate females. The results suggest a lack of inbreeding avoidance in the white rhinoceros. This conclusion is further supported by the fact that males mated with females that were, on average, more closely related to them than to the average pool of available females. Additionally, out of the two daughters that reached adulthood during the study period, one seemed to have mated successfully with her father (“A”). This finding constitutes the second report of possible incest in a white rhinoceros population (Guerier et al., 2012). Together, these results strongly suggest that white rhinoceros do not avoid mating with close relatives. Sex‐biased dispersal, a behaviour, which is widespread among mammals and birds (Pusey, 1987; Pusey & Wolf, 1996) and which has been observed in rhinoceros (Shrader & Owen‐Smith, 2002) may have been sufficient in historic times to limit mating with close relatives. Under the current situation, however, the lack of inbreeding avoidance could represent a severe threat for small and isolated populations of white rhinoceros. Inbreeding depression, which is common in many other species including various ungulate species (Ralls, Brugger, & Ballou, 1979), has not been described in white rhinoceros yet. It is likely that the negative effects, leading to juvenile mortality and abortions, may remain undetected in free‐ranging populations, in particular where predators are present.

### 4.1 Limits of the current study

One limitation of this study is the relatively poor assignment rate during paternity analyses. It was likely caused by the low genetic diversity in this species (Guerier et al., 2012), which decreases the power of genetic markers for pedigree reconstruction (Olsen, Busack, Britt, & Bentzen, 2001). Despite this limitation, we still managed to assign 73 juveniles out of 104 known mother–calf relationships, which represents the largest paternity analysis ever performed for any species of rhinoceros.

Likelihood‐based analyses can lead to a bias in paternity assignments mainly in favour of males that are unrelated to the females (Wang, 2010b). Our results do suggest that such a bias is unlikely to influence our study for several reasons. First, the presence of a strong bias would not be compatible with the finding that the males mated with females that were, on average, slightly more closely related to them than the average pool of available females. Second, we observed a relatively strong positive correlation between the relatedness and the mating or reproductive success of C1 males, which again is not consistent with the idea of a strong bias. Third, the alleles of the most successful males were not much different compared to the other males, so variation in mating and reproductive success is unlikely to stem from a bias in our paternity assignment. Finally, the metric we used to assess relatedness (DyadML) is not part of the list of relatedness estimators for which this problem has so far been demonstrated (Lynch & Ritland, 1999; Ritland, 1996; Wang, 2010b); nevertheless, the reliability of the metric remains to be studied in this particular context.

We used the 10 most polymorphic markers available in the species out of 60 tested (Scott, 2008), yet even those showed relatively low polymorphism (polymorphic information content <0.5) which is similar to that reported in another white rhinoceros population (Guerier et al., 2012). Our study covered two to four alleles per locus, which is below of what has been achieved in other studies (Guichoux et al., 2011), but our simulations show that our method of assignments was still sufficiently reliable in this condition. We were indeed able to compensate the relatively poor assignment success by including data known from the field observation: the mother–offspring relationships and the presence or absence of males within the pool of potential fathers. Our parentage assignment scheme resulted in a rate of correct assignment of 87%, which was much higher than what we obtained when not all field observations were considered. This study should be repeated using more polymorphic genetic markers. An ideal solution would be to develop a single nucleotide polymorphism (SNP) array based on full‐genome sequencing (Liu, Chen, Wang, Oh, & Zhao, 2005).

The largest limitation of our study is the low number of territorial males included in this study, which lead to a low statistical power of all correlation tests involving males. Unfortunately, such restriction is characteristic of remaining rhinoceros populations. In fact, our results are derived from one of the largest breeding populations in Southern Africa and from 13 years of paternity data. Moreover, the access to two independent sets of adult males allowed us to cross‐validate the possible trends identified within a given cohort. The results of such a cross‐validation suggest that none of the traits we analysed is a reliable predictor of the mating or reproductive success in male white rhinoceros. It is possible that other factors, which have not been included in this study, are responsible for the differences. The number of waterholes in a territory, the grazing value of a grass species and its nutritional value are possibilities. However, the number of traits analysed in this study was already high and the inclusion of additional habitat or food characteristics would have further reduced the statistical power. Ideally, the study should be repeated in a population that includes a higher number of territorial males, with the collection of a larger number of male and habitat characteristics. The best suited population meeting this characteristic is the free‐ranging population of the Hluhluwe–IMfolozi National Park. The problem there is that rhinoceros are not individually marked and genetic samples of all individuals are very difficult to collect.

An additional limitation of our study is that subadult and young adult males were absent for long time periods as part of the management intervention (with the exception of eight subadult males that were present between 1997 and 1998 and a young male number “60” who was present from 2001 until 2003). A possible consequence of the removal of subadults might be that the competition for territory ownership was reduced. However, two males were killed by territorial males during the study period (male “123” and male “60”) and one male was chased out of his territory (male “S”). We therefore believe that competition was high in our population. We also believe that the removal of subadults resembles a natural situation where subadults disperse out of the home range of territorial males (Owen‐Smith, 1975; Shrader & Owen‐Smith, 2002) and that male–male competition is particularly strong in fenced reserves, wherein rhinoceros have limited chance to disperse elsewhere as a strategy of competition avoidance. Again, it would be really interesting to replicate our study in a large unmanaged population representing natural levels of male–male competition, but such populations no longer exist. Even the founder population of all current rhinoceros (Hluhluwe–IMfolozi Park) or the very large Kruger National Park population are continuously managed (Beer, 2018).

### 4.2 Conservation management

The continuing fragmentation of habitats worldwide requires active management of the remaining herbivore populations in order to preserve them for coming generations (Ralls et al., 2018). Management measures usually involve antipoaching, culling, health care and disease control (Giglio et al., 2016). The preservation of the genetic variation, which is recommended by the IUCN and required by legislation in many countries, is only rarely put into practice (Ralls et al., 2018). One of the reasons for this lack in implementation could be that baseline data, such as the reproductive behaviour of a species, are not available. Many declining herbivore species are still poorly known and require basic research (Ripple et al., 2015). Even in a charismatic and well‐studied species such as the white rhinoceros, the breeding behaviour and the resulting effect on the genetic diversity of the species have not been studied extensively. The skew in reproductive success among territorial males, the high number of monogamous females in the population and the lack of incest avoidance among mating partners all lead to a reduction in genetic diversity in populations where dispersal and immigration are missing. In combination with intensive protection against poaching, the management of the genetic diversity of the white rhinoceros should therefore become a management priority, both in private hands and in national parks. Guidelines for translocation and reintroduction of rhinoceros already exist (Emslie et al., 2009; Emslie & Brooks, 1999) and should be expanded to incorporate our new findings.

There are a number of strategies that can be used to increase genetic variation in isolated populations. When pedigree information is available, management option could aim to selectively remove monogamous females and males that sired many offspring, or they could aim to retain individuals with specific rare alleles in the population.

When genetic data are not available, the introduction of individuals from outside the population becomes a popular tool (Bouzat et al., 2009) in order to increase genetic variation and fitness of small populations (Tallmon, Luikart, & Waples, 2004). In our study population, all territorial males were exchanged every 10 years to avoid inbreeding. Nevertheless, we still document a case of incest, which indicates that the 10 year time period was already too long and should be shortened to 6 years, which represents the time when young females reach sexual maturity (Owen‐Smith, 1975). We were not able to identify any environmental factor, which clearly influences the reproductive success in the white rhinoceros. Nevertheless, knowledge about such traits would provide game managers with simple tools to influence mate preference. For example, the supplement of water or food in certain areas can change the habitat quality (Cinková, Ganslosser, & Kretzschmar, 2017) and thus potentially female mate choice and reproductive success of individual males. We therefore urge further studies in this direction. A video abstract can be viewed here.

## CONFLICT OF INTEREST

None declared.

## Data Availability

All R codes and the data are provided as a supplementary R package at https://github.com/courtiol/matingRhinos.
