# Supplementary material for: Mate choice, reproductive success and inbreeding in white rhinoceros: New insights for conservation management
Source: Evol Appl. 2019 Dec 16;13(4):699–714. doi: 10.1111/eva.12894 (PMC7086106; doi:10.1111/eva.12894)
Supplement: Supplementary file 1 [file EVA-13-699-s001.pdf]

Supplemental Information for:

Mate choice, reproductive success and inbreeding in white rhinoceros: New insights for conservation management.

Supplement 1

Transect points:

Transect points were located within a square grid of 0.8 minutes length, distributed over the entire study area. The points were located using a GPS, giving a ± 100 m deviation in distance between points. The survey was done in October 1998 and lasted three weeks. During this period rainfall was little (6.9 mm) and no fresh grass was observed.

Supplement 2

Food selection and volume of selected food:

For the food selection study we compared the number of times an individual grass species was available along a feeding path with the number of times this particular grass species has been eaten by a rhinoceros. This study is a preliminary analysis for the study on differences in habitat characteristics and was conducted on females only. For this, we followed the footprints of foraging females (n = 6) with the help of a local game tracker. Once a feeding event has been identified, such as by the number of tracks that were scattered within circles, the location of a feeding patch could be recognized by the characteristic fresh green bite marks on the top of a grass species. We placed a 1m x 1m plot on each feeding patch and identified each individual grass species therein. Once a certain grass species has been eaten within a feeding plot, it was counted as a single feeding event for this grass species.

Additionally, we established the availability of grass species along the feeding path by placing 1m x 1m plot along the feeding path approximately 100m apart from each other. Again all grass species within this plot were identified and the presence of a single species within each plot was counted as a single event. We carefully balanced the number of feeding and non-feeding plots within one tracking session in order to minimize autocorrelation. Data collection was terminated if no feeding occurred within a distance of 1 km, which resulted in a total of 91 feeding plots and 59 non-feeding plots.

To establish whether a particular grass species has been selected during feeding, all feeding events for this grass species were added and divided by the sum of all non-feeding events for this particular grass species. Finally, the grass species with the highest frequency has been used for the comparison of habitat characteristics within male territories.

We estimated the volume of selected grass species within male territories using four 1m x 1m plots placed in the four directions of the compass, ten meters apart from the main transect point. We herewith accounted for heterogeneous vegetation and included a larger area in the transect analysis. Within each plot, we estimated the presence of individual grass species, their cover

(Greig-Smith 1957) and average height. We then calculated the mean volume of all plots and all transect points.

Supplement 3

Microsatellite loci:
Each locus possessed 2-4 alleles with frequency skewed towards one allele. Only SR63 had equal weighting to the two alleles present at that locus. There was a presence of unique alleles at six of the 11 loci (BR06, DB44, WR32A, WR32F, WR32A, SR54). Four individuals in the parent pool were not typed at SR63 (1 breeding female, 3 males), one male at WR32F and one male at SR262, resulting in a white rhinoceros being genotyped at an average of 98.01% of the 22 potential alleles. We found no evidence of null alleles or stuttering for the final 10 loci used in this study. Mean probability of non-exclusion of the true father given the known mother was 0.333 for the 11 loci, and 0.389 when WR32F was excluded. The highest non-exclusion probability occurred at locus SR262 and the lowest at locus SR281.

Table S1: **Summary statistics for the microsatellite loci used in this study**. Primer sequence, publication source, the number of alleles per locus (k), the number of samples (N), the observed (*H_{obs}*) and expected heterozygosity (*H_{exp}*), the polymorphism information content (*PIC*), non-exclusion probability of the father given the known mother (*NE-1P*) and the individual genotyping error for 11 microsatellite loci. The locus WR32F deviated significantly from Hardy Weinberg Expectations.

loci	primer sequence (5'-3')	source	k	N	H_{obs}	H_{exp}	PIC	NE-1P	genotyping error
BR06	F: TCATTTCTTTGTTCCCCATAGCAC<br>R: AGCAATATCCACGATATGTGAAGG	Cunningham et al. 1999	4	50	0.60	0.482	0.433	0.8814	0.07
DB01	F: AGATAATAATAGGACCCTGCTCCC<br>R: GAGGGTTTATTGTGAATGAGGC	Brown & Houlden 1999	2	50	0.28	0.272	0.233		0.03
DB44	F: GGTGGAATGTCAAGTAGCGG<br>R: CTTGTTGCCCCATCCCTG	Brown & Houlden 1999	3	50	0.44	0.388	0.339	0.9263	0.02
WR32A	F: CAGTCCTGCTGCATAAATCTC<br>R: GCAGTACAGCTAGAATCAC	Florescu et al. 2003	3	50	0.58	0.524	0.403	0.8655	0.09
WR32F	F: AATACTCAAGCTATGCATCC<br>R: TGTCGTCTACATATAGGGT	Florescu et al. 2003	3	50	0.72	0.543	0.437	0.8567	0.03
WR35A	F: AGCCTGCTTTGCTGCCTTGC<br>R: AGGTGCACACATCCCACTCG	Scott 2008	4	50	0.28	0.388	0.325	0.9259	0.03
IR12	F: GAATGCTGATCATTTAGTGAC<br>R: GGGTCCAGTTGAGATATCAC	Scott 2008	2	50	0.28	0.389	0.311	0.926	0.03
SR54	F: CAATATCCAGGCTTCCAGG<br>R: CTGTTTACTGTTATCGATGCT	Scott et al. 2004	3	50	0.20	0.216	0.195	0.9772	0.03
SR63	F: CTTGAGCAGAGTAGAATTTGG<br>R: CTCTGTATCCACCTCATTCC	Scott et al. 2004	2	46	0.52	0.505	0.375	0.875	0.05
SR262	F: CTGCCTTAACAACTGAACTGC<br>R: TGGAGGTTATCTCATGCCAC	Scott 2008	2	49	0.31	0.262	0.226	0.9664	0.02
SR281	F: AGGTGATTAGGGAATTGCTGG<br>R: TTCTTCTGTCCTGGCATTGC	Scott et al. 2004	3	50	0.50	0.629	0.552	0.8058	0.06

Table S2: **The number of offspring allocated with COLONY to reproductive active female rhinoceros and the two cohorts of males including the two additional males: ‘60’ and ‘123’.**

	**Cohort 1 males**							**Cohort 2 males**							
**Mother**	**A**	**G**	**K**	**R**	**S**	**123**	**total**	**5**	**30**	**60**	**62**	**63**	**65**	**66**	**total**
**3**	1						1						1		1
**4**					1		1								0
**7**	1*						1								0
**8**	1						1								0
**9**			1				1	2							2
**11**	1						1		1						1
**15**					1		1	1			1	1			3
**17**	1						1					1			1
**21**					1		1						1		1
**23**	1						1	2							2
**24**			1				1		1						1
**27**		1					1								0
**33**		3					3					1			1
**34**	1						1								0
**35**		1		1			2								0
**37**	1						1				1				1
**38**							0		1			1	1		3
**41**			1				1						1		1
**43**			1				1	1							1
**45**	1						1		1						1
**48**							0						1		1
**53**			3				3	1					1		2
**54**			1				1		1		1				2
**56**				1			1	2							2
**58**			1				1				1				1
**60**			3				3	1					1		2
**61**				1			1						1		1
**67**	too young to breed with C1 ♂						0						2		2
**78**				2			2						1		1
**81**	too young to breed with C1 ♂						0		1						1
**91**	too young to breed with C1 ♂						0				1		1		2
**97**	too young to breed with C1 ♂						0						1		1
**118**									1						1
**total**	9	5	12	5	3	0	**34**	10	6	0	5	4	13	0	**39**

* mating with daughter

Table S3: **The number of transect points, the availability of the four habitat types, the availability of selected grass species in the territories and the average volume of selected grass species within the territories of cohort 1 and cohort 2 males.** The assessment was done at the end of the dry season.

	**cohort 1**					**cohort 2**					
	**A**	**G**	**K**	**R**	**S**	**5**	**30**	**62**	**63**	**65**	**66**
**Transect points**	34	27	33	40	4	32	29	9	38	26	12
**Habitat openness [%]**											
'grassland'	18	0	6	23	0	3	10	11	0	0	0
'open woodland'	27	26	42	32	0	44	52	22	26	19	83
'close woodland'	38	41	46	38	50	47	31	45	37	58	17
'thickets'	18	33	6	8	50	6	7	22	37	23	**0**
**Average occurrence of selected grass species [%]**											
Panicum maximum	45	60	64	56	46	63	41	67	58	58	16
**Average volume of selected grass species within male territories [m^3]**											
Panicum maximum	4.44	2.96	2.88	2.54	0.85	1.34	2.33	2.07	1.91	4.56	0.6

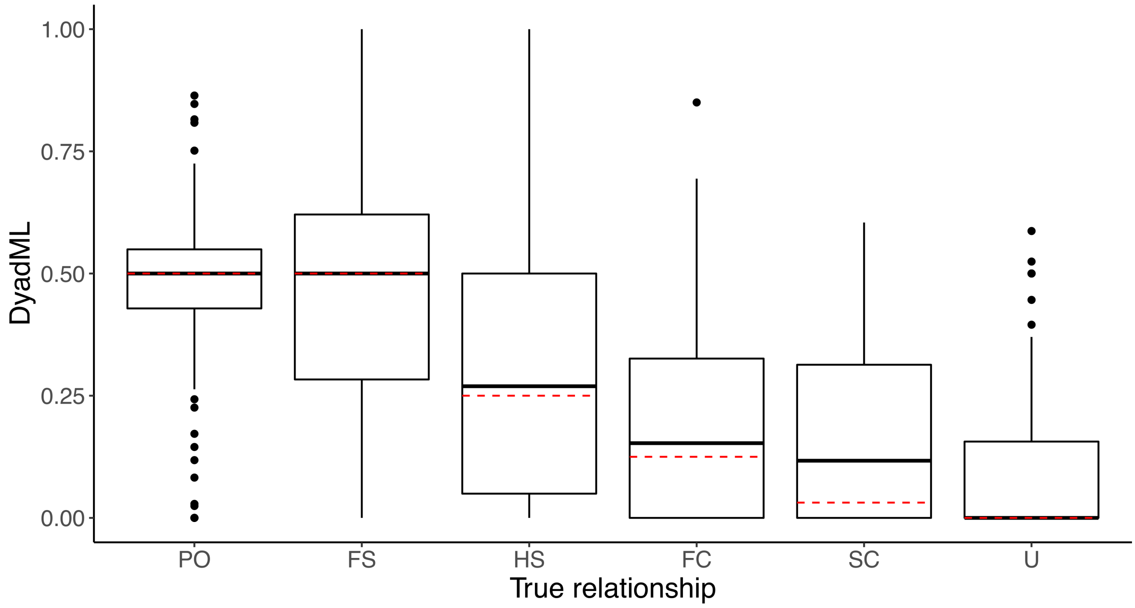

Figure S1: **Performance of the DyadML estimator of relatedness assessed by simulation.** This figure shows relatedness coefficients estimated by DyadML in COANCESTRY for 100 simulated dyads in six relatedness categories. The simulations of relatedness were performed using the allele frequencies of all parents and offspring in our population. Boxes represent the first and third quartiles and the bold horizontal line indicates the median. The vertical lines extend to the most extreme observations no further than +/- 1.5 times the interquartile range from the closest hinge of the box. The dots represent the outliers. The dashed red lines mark true coefficients of 0.5 (for parents-offspring (PO) and full siblings (FS)), 0.25 (half siblings/grandparent-grandchild (HS)), 0.125 (first cousin (FC)), 0.01325 (second cousin (SC)) and 0 (unrelated (U)).

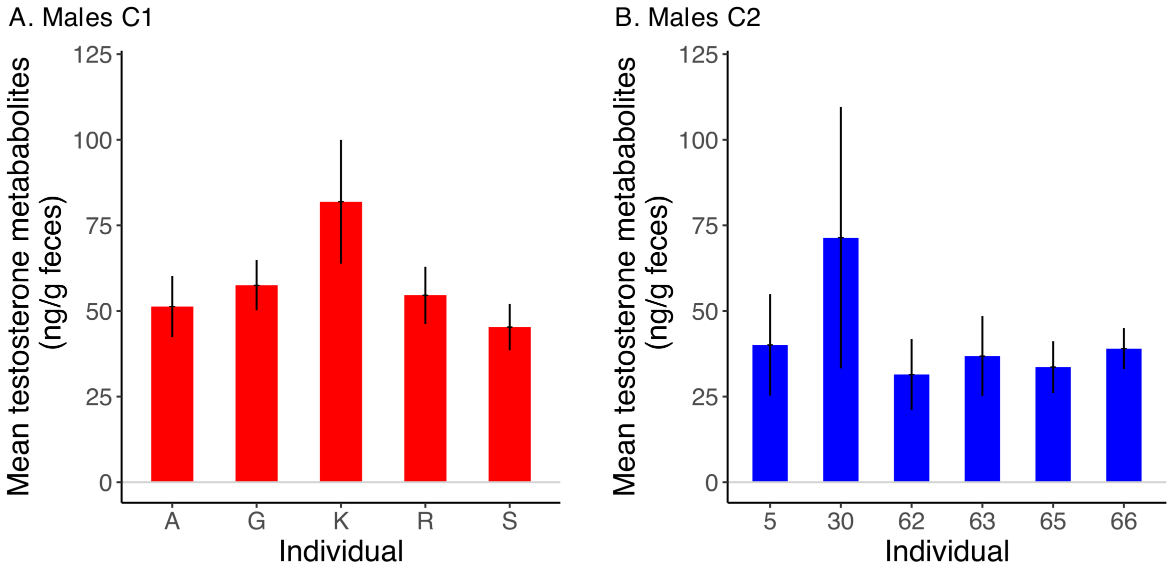

Figure S2: **The testosterone metabolites concentration measured in faecal samples from cohort 1 (n = 59 ± 6 samples/male) and cohort 2 males (n = 5 ± 0.4 samples/male).** Values in the figure are means ± standard deviation.

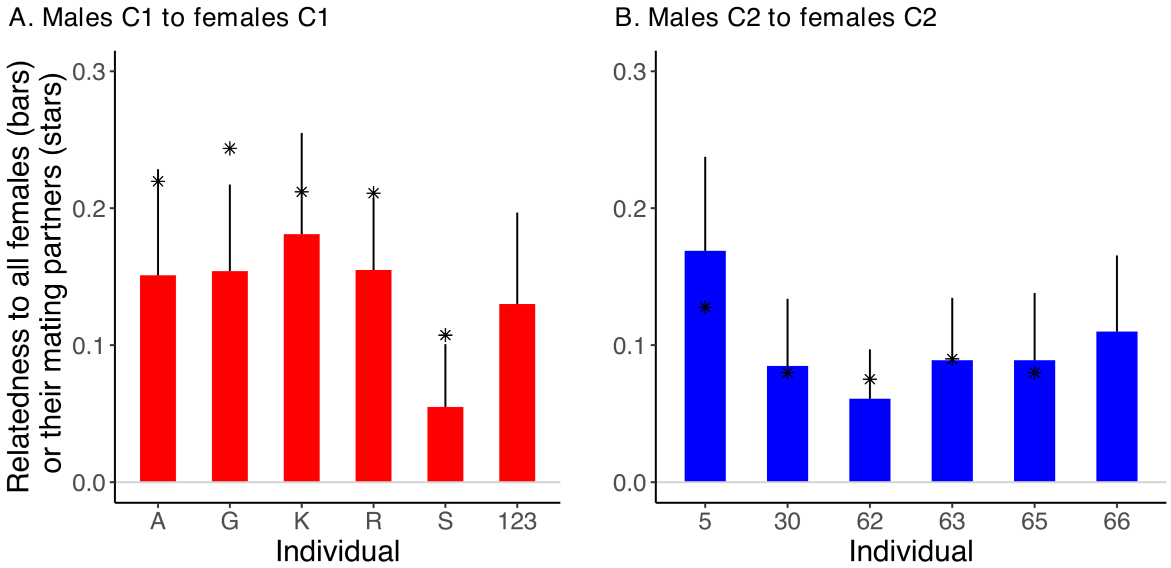

Figure S3: **The degree of mean relatedness as estimated by the maximum likelihood estimator DyadML in COANCESTRY between A) each male of cohort 1 and all reproductively active females and B) each male of cohort 2 and all reproductively active females.** Values in the graph are means ± standard deviation. The star indicates the mean relatedness of males with their mating partners.
